# Anterior Chamber Migration of Ozurdex Implants

**DOI:** 10.4274/tjo.galenos.2019.43778

**Published:** 2020-04-29

**Authors:** Özcan Kayıkçıoğlu, Suzan Doğruya, Cansu Sarıgül, Hüseyin Mayalı, Emin Kurt

**Affiliations:** 1 Celal Bayar University Faculty of Medicine, Department of Ophthalmology, Manisa, Turkey; 2 Uşak University Training and Research Hospital, Clinic of Ophthalmology, Uşak, Turkey

**Keywords:** Dexamethasone implant, phacoemulsification, corneal edema, pars plana vitrectomy

## Abstract

We present patient characteristics and follow-up results of cases with anterior chamber dexamethasone implant migration. The common feature of all six presented cases was vitrectomized eyes. Four of the patients had sutured intraocular lens (IOL) implantation due to complicated cataract surgery, one had combined retinal detachment surgery with sutured IOL implantation, and one had vitrectomy for diabetic intravitreal hemorrhage cleaning and uncomplicated cataract surgery. Anterior chamber implant migration caused corneal edema in all cases and elevated intraocular pressure in three cases. In two cases, the dexamethasone implant was directed into the vitreous cavity after maximum pupillary dilation and corneal manipulation with cotton tip applicator with the patient in reverse Trendelenburg position. There was no other complication until dexamethasone implant degradation, with clear cornea at final examination. In two cases, the implant was removed from the anterior chamber by aspiration, but keratoplasty surgery was planned due to endothelial cell loss and persistent corneal edema during follow-up. In the last two cases, the dexamethasone implant was redirected into the vitreous chamber with a 23-gauge catheter and anterior chamber maintainer but they migrated into the anterior chamber again. In one of these patients, the implant was aspirated by catheter and corneal transplantation was performed due to corneal edema, while the other patient’s implant was redirected into the vitreous chamber with no further anterior migration. The risk of dexamethasone implants migrating into the anterior chamber of vitrectomized eyes and those with sutured IOL implantation should be kept in mind and the patient should be informed and advised to see an ophthalmologist immediately before permanent corneal endothelial damage occurs.

## Introduction

Ozurdex (Allergan Inc. Irvine, CA, USA) is a rod-shaped, biodegradable dexamethasone implant 6 mm in length and 0.46 mm in diameter that is injected into the intravitreal cavity using a 22-gauge needle. It is effective in the treatment of macular edema due to retinal vein occlusion, non-infectious uveitis affecting the posterior segment, and diabetic macular edema.^1,2,3^ After implantation, the Ozurdex polymer matrix releases 0.7 mg preservative-free dexamethasone into the intravitreal cavity and degrades into lactic acid and glycolic acid. The most common complication reported after dexamethasone implantation is an increase in intraocular pressure, which peaks at about 3 months.^4,5^ In addition to the side effects of dexamethasone reported in the literature, such as cataracts and increased intraocular pressure, the implantation procedure itself involves the risk of complications like dislocation to the anterior chamber, corneal endothelial damage, secondary corneal edema, and implantation in the lens.^6,7,8^ Migration of a dexamethasone implant into the anterior chamber is a rare complication that can be managed by directing the implant back into the vitreous cavity or removing it from the anterior chamber through a corneal incision.^7,9,10^ In this series of six cases, we discuss risk factors, clinical course, and treatment approaches for migration of dexamethasone implants to the anterior chamber.

## Case Report

Patients who underwent dexamethasone implantation in the ophthalmology department of Manisa Celal Bayar University Faculty of Medicine within the past 5 years and had anterior chamber dislocation of the implants during follow-up are discussed in terms of etiologies, treatment approaches, and outcomes.

### Case 1

**Figure 1:**
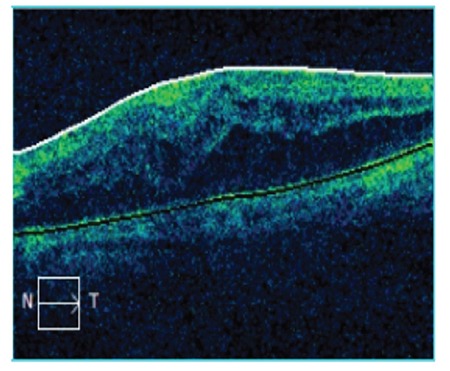
Follow-up OCT shows cystoid macular edema in a patient who underwent PPV with silicone oil injection due to left retinal detachment (Case 1) OCT: Optical coherence tomography, PPV: Pars plana vitrectomy

**Figure 2:**
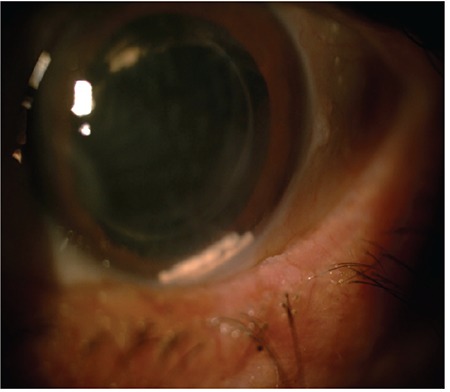
Two implants are observed in the anterior chamber of a patient who was given a dexamethasone implant due to post-vitrectomy macular edema and did not regularly attend follow-up visits (Case 1)

**Figure 3:**
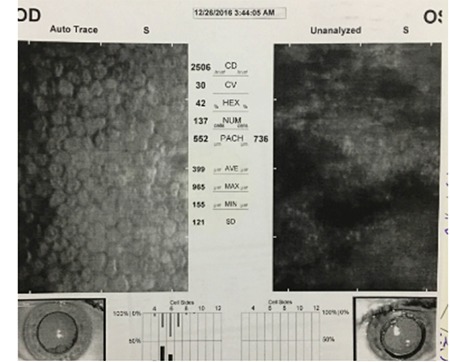
Follow-up specular microscopy reveals a decrease in corneal endothelial cell number after the removal of two dexamethasone implants from the anterior chamber (Case 1)

A 63-year-old man was being followed in our retina unit after undergoing pars plana vitrectomy (PPV) with silicone oil injection due to retinal detachment in the left eye, followed by silicone removal 4 months later. At last examination, his Snellen visual acuity was 0.7 in the right eye and 0.15 in the left eye. Intraocular pressure (IOP) was 14 mmHg in the right eye and 16 mmHg in the left eye. On slit-lamp examination, nuclear cataract was observed in the right eye, while pseudophakia, posterior capsule defect, and zonular dialysis were observed in the left eye. Fundus examination was normal in the right eye and showed attached retina and peripheral cryotherapy and laser scars in the left eye. On optical coherence tomography (OCT) imaging, the right eye appeared normal, while cystoid macular edema (central macular thickness: 577 µm) was observed in the left eye (Figure 1). Fundus fluorescein angiography (FFA) imaging revealed diffuse hyperfluorescence consistent with macular edema. The patient received a dexamethasone implant for post-vitrectomy macular edema. At 1-week follow-up after implantation, severe corneal edema was observed on slit-lamp examination. IOP was measured as 42 mmHg. After lowering his IOP to within normal range with medical treatment, two dexamethasone implants were observed situated at the angle in the anterior chamber (Figure 2). Both implants were removed from the anterior chamber by aspirating with a 23-gauge (G) catheter. Both were found to be their original size and were evaluated as newly implanted. There was no record of the administration of a second implant in our clinic, and sufficient information could not be obtained from the patient regarding the second implant. He reported that he was also being followed up by other ophthalmologists at another center. At 6-month follow-up, he exhibited bullous keratopathy and irreversible permanent endothelial damage. He was evaluated in the cornea unit and keratoplasty was planned (Figure 3).

### Case 2

**Figure 4:**
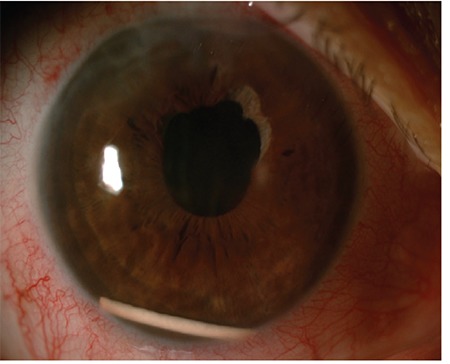
A dexamethasone implant given to treat macular edema in a patient who underwent PPV with sutured IOL implantation after cataract surgery is detected in the anterior chamber (Case 2) PPV: Pars plana vitrectomy, IOL: Intraocular lens

A 60-year-old woman who was referred to our clinic from another center due to complicated cataract surgery underwent PPV and sutured intraocular lens (IOL) implantation. In the last examination, her Snellen visual acuity was 0.1 in the right eye and 1.0 in the left eye, while IOP was 19 mmHg in the right eye and 18 mmHg in the left eye. On fundus examination, the macula was elevated and optic disc was normal in the right eye, while the left eye was entirely normal. OCT revealed cystoid edema in the right eye and normal findings in the left eye. FFA imaging in the right eye showed late-phase hyperfluorescence in the macula, and it was decided to treat her right eye with dexamethasone implant. In examination performed 2 weeks after implantation, the right eye exhibited mild corneal edema and the dexamethasone implant was visible in the anterior chamber (Figure 4). Fundus examination showed retinal attachment. After inducing pupil dilation, the patient was placed in the reverse Trendelenburg position and the implant was directed into the vitreous cavity under topical anesthesia by corneal manipulation with a sterile cotton tip applicator. After mydriasis subsided, the patient was advised to avoid bending over forward and to sleep in a 45-degree upright position until the implant degraded. At follow-up examination, clear cornea and calm anterior chamber were observed on slit-lamp examination of the patient’s right eye. On fundus examination, the retina was attached and the dexamethasone implant was observed in the inferior hemisphere. The implant did not appear in the anterior chamber again before dissolving over the follow-up period of 5 months.

### Case 3

**Figure 5:**
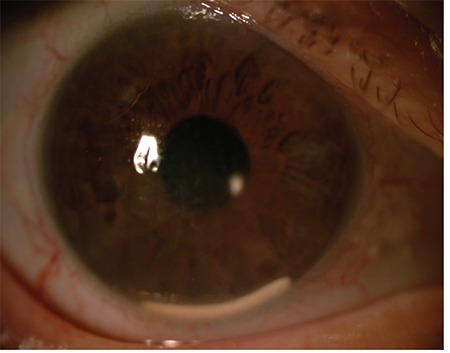
A dexamethasone implant given to treat macular edema in a patient with intravitreal hemorrhage due to proliferative diabetic retinopathy who underwent PPV and endolaser therapy is seen in the anterior chamber (Case 3) PPV: Pars plana vitrectomy

**Figure 6:**
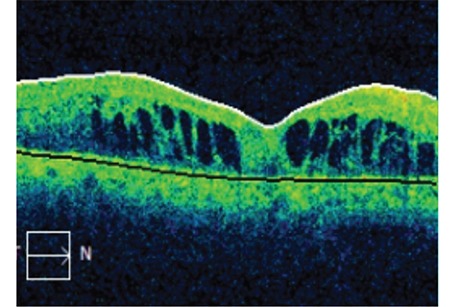
OCT shows persistent macular edema in a patient given dexamethasone implants to treat macular edema due to proliferative diabetic retinopathy (Case 3) OCT: Optical coherence tomography

**Figure 7:**
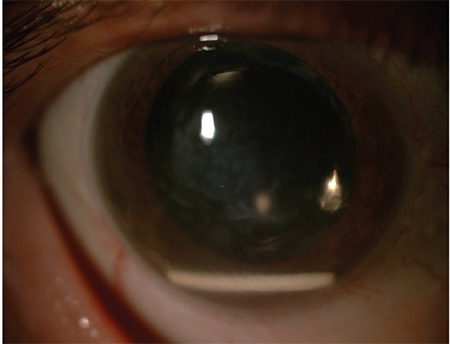
Anterior chamber migration of the implant was observed at follow-up in a patient who received a third dexamethasone implant due to macular edema (Case 3)

A 61-year-old man with proliferative diabetic retinopathy who underwent PPV, endolaser, and phacoemulsification with IOL implantation in the right eye and PPV and endolaser in the left eye due to bilateral intravitreal hemorrhage was being followed up in our retina unit. At last examination, visual acuity was 0.05 cm in the right eye and counting fingers at 50 cm in the left eye, while intraocular pressure was 17 mmHg in the right eye and 16 mmHg in the left eye. Slit-lamp examination revealed pseudophakia and clear cornea in the right eye and posterior subcapsular cataract in the left eye. On fundus examination, cellophane maculopathy, laser scars in the periphery, and macular edema were observed in the right eye. Laser scars and macular microhemorrhages were observed in the left eye. Bilateral cystoid macular edema was noted on OCT. No response was achieved despite 4 bilateral intravitreal ranibizumab injections, so the decision was made to administer dexamethasone implants. Slit-lamp examination at 1-month follow-up after dexamethasone implantation revealed corneal edema and the dexamethasone implant was seen in the anterior chamber of the right eye (Figure 5). Using a 23-G catheter and anterior chamber maintainer, the implant was moved from the anterior chamber and directed to the vitreous cavity through the zonular area. At follow-up 9 months after implantation, an increase in cystoid macular edema was observed and the patient was given a second dexamethasone implant. No complications related to the dexamethasone implant were noted during follow-up. Due to clinical and OCT findings of recurrent cystoid macular edema at 7-month follow-up, a third dexamethasone implant was administered (Figure 6). At follow-up examination 2 weeks after implantation, the cornea exhibited edema and bullae, and the dexamethasone implant was again seen in the anterior chamber (Figure 7). Using a 23-G catheter and anterior chamber maintainer, the implant was removed from the anterior chamber and repositioned in the vitreous cavity. Regression of the corneal edema was observed on follow-up examination.

### Case 4

**Figure 8:**
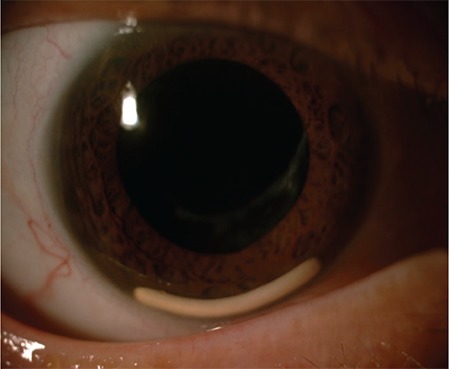
A dexamethasone implant given to treat macular edema in a patient who underwent PPV with sutured IOL implantation after complicated cataract surgery is observed in the anterior chamber (Case 4) PPV: Pars plana vitrectomy, IOL: Intraocular lens

**Figure 9:**
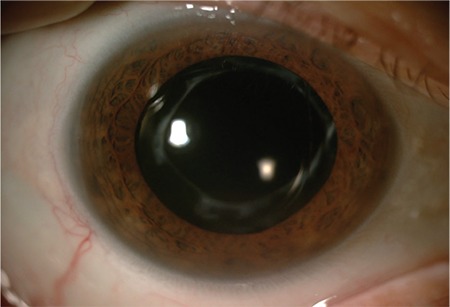
Clear cornea is observed at follow-up after guiding the dexamethasone implant from the anterior chamber back into the vitreous cavity (Case 4)

A 79-year-old woman who was referred to our clinic from another center for complicated cataract surgery underwent left PPV and sutured IOL implantation. At last examination, her Snellen visual acuity was 0.6 in the right eye and counting fingers at 3 m in the left eye; IOP was 16 mmHg in the right eye and 14 mmHg in the left eye. Both eyes were found to be pseudophakic on slit-lamp examination. Fundus examination revealed irregularity of the retinal pigment epithelium in the right eye and laser scars in the inferior hemisphere of the left eye. OCT findings were normal in the right eye, while spongy edema was observed in the left eye. FFA imaging showed macular edema with traction in the inferotemporal quadrant of the left eye, and it was decided to administer a dexamethasone implantation. The implant appeared in the anterior chamber 15 days later (Figure 8). After pupillary dilation, the patient was placed in the reverse Trendelenburg position and the implant was directed into the vitreous cavity by corneal manipulation with a sterile cotton tip applicator. After mydriasis subsided, the patient was advised to avoid bending over forward and to sleep in a 45-degree upright position until the implant degraded. At follow-up examination, the dexamethasone implant had degraded and the cornea was clear (Figure 9).

### Case 5

**Figure 10:**
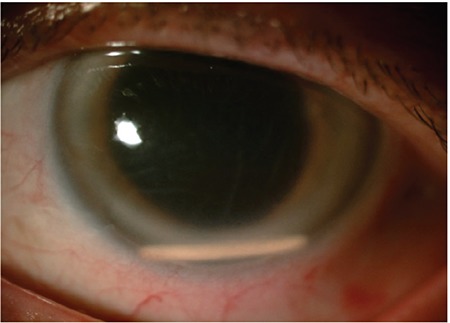
A dexamethasone implant given to treat macular edema in a patient who underwent PPV with scleral-fixated IOL implantation due to traumatic cataract is seen in the anterior chamber (Case 5) PPV: Pars plana vitrectomy, IOL: Intraocular lens

**Figure 11:**
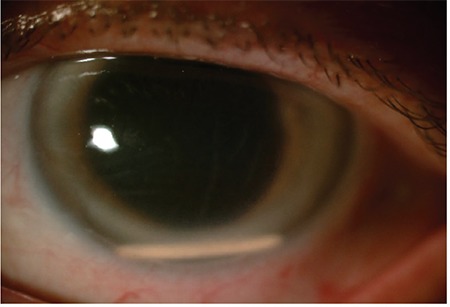
The dexamethasone implant migrated to the anterior chamber again the day after being repositioned in the vitreous cavity using 23-gauge catheter (Case 5)

**Figure 12:**
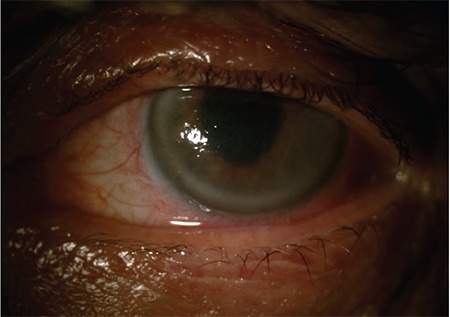
Bullous keratopathy is observed at follow-up after the dexamethasone implant that migrated to the anterior chamber twice was removed with a 23-gauge catheter (Case 5)

A 73-year-old man who was referred to our clinic from another center due to traumatic cataract and zonular dialysis underwent left PPV and anterior vitrectomy, followed by scleral-fixated IOL implantation 2 months later. In ophthalmologic examination at postoperative 2 months, his Snellen visual acuity was 0.9 in the right eye and counting fingers at 1 m in the left eye, while IOP was 12 mmHg in the right eye and 18 mmHg in the left eye. Both eyes appeared pseudophakic on slit-lamp examination. Fundus examination revealed macular retinal pigment epithelial irregularity in the macula in both eyes. On OCT, the right eye was normal, while macular edema (447 µm) was observed in the left eye. The right eye showed no leakage on FFA imaging, while the left eye showed macular edema and areas of hyperfluorescence. A dexamethasone implant was injected into the left eye. The patient presented to the clinic 25 days after implantation with further visual deterioration (hand movements). The implant was observed in the anterior chamber of the left eye on slit-lamp examination (Figure 10). Using a 23-G catheter and anterior chamber maintainer, the implant was removed from the anterior chamber and repositioned in the vitreous cavity. However, the dexamethasone implant appeared in the anterior chamber again the next day (Figure 11). The implant was removed from the anterior chamber by aspiration with a 23-G catheter. Follow-up examinations revealed corneal edema and visual acuity of counting fingers at 10 cm in the left eye. Upon development of bullous keratopathy, the patient was evaluated in the cornea unit and underwent keratoplasty 8 months later (Figure 12).

### Case 6

**Figure 13:**
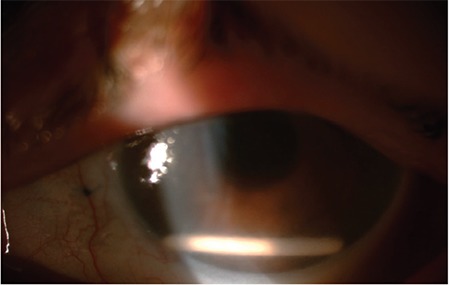
Anterior chamber migration of a dexamethasone implant given to treat macular edema is observed in a patient who underwent pars plana vitrectomy and scleral-fixated IOL implantation (Case 6) IOL: Intraocular lens

A 70-year-old man who was being followed up in our clinic for angle-closure glaucoma underwent left PPV and phacoemulsification, followed by scleral-fixated IOL implantation 6 months later. At last examination, his Snellen visual acuity was 1.0 in the right eye and 0.2 in the left eye; IOP was 12 mmHg in the right eye and 17 mmHg in the left eye. On slit-lamp examination, shallow anterior chamber and patent iridotomy were observed in the right eye and clear cornea, iridotomy, centered IOL, and iris atrophy in the superior quadrant were observed in the left eye. On fundus examination, cup-to-disc ratio was normal in the right eye and 0.3 in the left eye. OCT findings were normal in the right eye, while macular edema (434 µm) was present in the left eye. On FFA imaging, there was no leakage in the right eye, while the left eye showed macular edema with late-phase hyperfluorescent areas. The patient received 5 intravitreal ranibizumab injections. Due to macular edema (550 µm), it was decided to administer a dexamethasone implant. Twenty days after implantation, the patient presented to the clinic with complaints of vision loss. His visual acuity was at the level of hand movements in the left eye, and slit-lamp examination revealed corneal edema and the dexamethasone implant in the anterior chamber (Figure 13). His IOP was 22 mmHg with topical and systemic treatment. The implant was removed by aspiration with a 23-G catheter. In follow-up examinations, corneal edema and bullous keratopathy were detected in his left eye. He was assessed in the cornea unit and keratoplasty was planned.

The characteristics and outcomes of patients with dexamethasone implant migration from the vitreous cavity to the anterior chamber are presented in Table 1.

## Discussion

In this study, we present information pertaining to patients who were followed up and treated for dislocation of dexamethasone implants into the anterior chamber. The patients included 3 women and 3 men between the ages of 60 and 79 years. All of the affected eyes were vitrectomized and pseudophakic. One patient (Case 1) had posterior capsule defect, zonular dialysis, and IOL implantation in the sulcus, 4 patients (Cases 2, 4, 5, and 6) had undergone sutured IOL implantation after complicated cataract surgery, and 1 patient (Case 3) had undergone intracapsular IOL implantation with no posterior capsule defect. Anterior chamber migration of a dexamethasone implant is a rare complication and risk factors include previous vitrectomy, aphakic eyes, posterior capsule opening, and lying in prone position.^7,11^ Long plane journeys within the first week after dexamethasone implantation were also reported to be a risk factor for anterior chamber dislocation by increasing vitreous pressure due to changes in air pressure.^12^ The clinical findings and risk factors of our patients were consistent with the information in the literature. All of our patients had undergone PPV and had posterior capsule defect and/or zonular dialysis.

In the event of a dexamethasone implant in the anterior chamber, the implant can be removed from the anterior chamber via a corneal incision or directed back into the vitreous cavity.^13^ In addition, spontaneous return of the implant to the vitreous cavity has also been reported.^14^ The procedure of guiding the implant back into the vitreous cavity by placing the patient in supine position after pupil dilation was first described by Kishore and Schaal.^9^ Mateo et al.^15^ reported scleral fixation of the dexamethasone implant using a 10-0 suture. In 2 of our patients with dexamethasone implant in the anterior chamber (patients 2 and 4), the patients were placed in reverse Trendelenburg position after pharmacological dilation and the implant was guided back into the vitreous cavity by manipulating the cornea with a sterile cotton tip applicator. In 2 other patients (patient 3 and patient 5 after first migration), the implant was moved from the anterior chamber back into the vitreous cavity using a 23-G catheter and anterior chamber maintainer. In the patients with severe corneal edema and elevated IOP (patients 1, 6, and 5 after second migration), the dexamethasone implant was removed from the anterior chamber using a 23-G catheter. One of the 2 dexamethasone implants detected in the anterior chamber in patient 1 was implanted in our center, while the other was most likely implanted as a second dose within a short period at another center. This could not be explained conclusively.

Khurana et al.^7^ reported that corneal edema developed when anterior chamber migration occurred within the first 3 weeks after dexamethasone implantation, but did develop in migrations occurring between 5 weeks and 3 months after implantation. All of the patients in our series presented with early migration, within 1 month of implantation, and severe corneal edema was observed in 3 of the patients (patients 1, 5, and 6). Keratoplasty was planned for patient 6 and patient 1, who had 2 implants in the anterior chamber, due to persistent bullous corneal changes and very low endothelial cell counts. Patient 5 underwent keratoplasty due to bullous keratopathy and corneal endothelial failure. The patients who did not develop corneal edema were those who presented to our clinic promptly after onset of their complaints and received rapid intervention.

Kang et al.^16^ retrospectively analyzed 924 cases of intravitreal dexamethasone injection. Anterior chamber migration of the implant occurred in 4 patients within 2 to 6 weeks. In 2 patients, the implant was guided back into the vitreous cavity. In the other 2 patients, implant migration occured twice in one and 3 times in the other before the implants were surgically removed. One of the patients underwent keratoplasty. All of the affected eyes lacked posterior capsule integrity.^16^ In our case series, 2 dexamethasone implants were detected in the anterior chamber of 1 patient (Case 1). They were removed from the anterior chamber by aspiration using a 23-gauge catheter. Keratoplasty was planned for this patient in the cornea unit because he developed bullous keratopathy and corneal endothelial decompensation. In the case of 2 other patients (Cases 2 and 4), the implant was maneuvered back into the vitreous cavity by applying pressure to the cornea with a sterile cotton tip applicator with the patient in supine position after pupil dilation and reverse Trendelenburg positioning. This resulted in no further problems until implant degradation and the cornea was clear at final examination. In 2 of our patients (Cases 3 and 5), the implant was removed from the anterior chamber and surgically repositioned in the vitreous cavity using a 23-G catheter and anterior chamber maintainer. However, in both patients, repeat migration of the dexamethasone implant was observed. In 1 of these patients (Case 3), the implant was surgically repositioned again with no further problems, while in the other patient (Case 5) explantation was performed using a 23-G catheter. This patient later underwent keratoplasty due to corneal endothelial failure and bullous keratopathy. The implant was also explanted from another patient (Case 6) using a 23-G catheter. Keratoplasty was planned in the cornea unit due to the development of corneal endothelial failure and bullous keratopathy. In all of our patients, the dexamethasone implant migrated into the anterior chamber within 1 to 4 weeks of implantation. Five patients (Cases 1, 2, 4, 5, and 6) lacked posterior capsule integrity and all patients had undergone PPV.

Goncalves et al.^17^ retrospectively analyzed 468 patients who received dexamethasone implant injections at multiple centers and determined the prevalence of implant migration to be 1.6%. They also reported a significant relationship between implant migration and cataract surgery (p=0.043), intraocular lens status (p=0.005), and vitrectomy (p=0.057). Öner et al.^18^ injected a dexamethasone implant for macular edema in a patient who underwent PPV and scleral-fixated IOL implantation after complicated cataract surgery. Fifteen days after the implantation, the patient exhibited corneal edema and anterior chamber migration of the dexamethasone implant. Explantation was performed, but corneal edema persisted at 4-month follow-up. All of our patients had history of cataract surgery and vitrectomy. Four had sutured intraocular lenses, 1 had an open posterior capsule, zonular dialysis, and a sulcus lens, and 3 patients developed bullous keratopathy.

In cases of anterior chamber dislocation of dexamethasone implants, the implant should be removed or repositioned in the vitreous cavity as soon as possible in order to prevent permanent corneal edema due to corneal endothelial damage. Pupil dilation and reverse Trendelenburg positioning followed by positional guidance of the implant toward the vitreous by cornea manipulation with a sterile cotton tip applicator is a noninvasive procedure that can be used as a first approach in suitable patients. Patients should be advised to avoid long trips and the prone position after dexamethasone implantation and to see an ophthalmologist immediately if they experience any ocular complaints.

Risk factors for anterior chamber migration of dexamethasone implant such as PPV, previous complicated cataract surgery, lack of posterior capsule integrity, and zonular dialysis should be evaluated carefully and implantation should be avoided in patients who are at risk.

## Figures and Tables

**Table 1:**
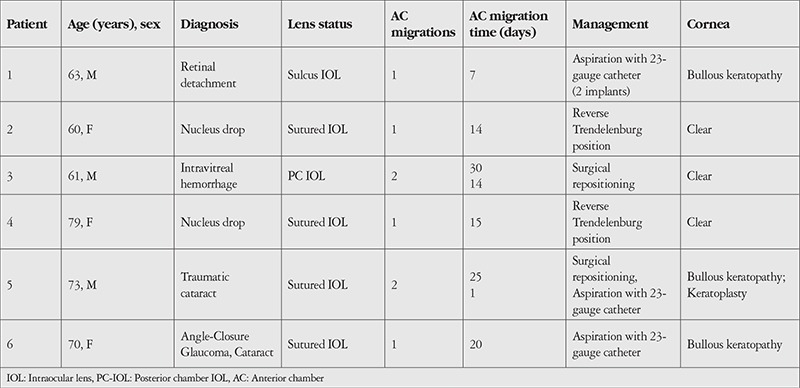
The characteristics and outcomes of patients with dexamethasone implant migration from the vitreous cavity to the anterior chamber
